# The Passive Leg Raising Test To Guide Fluid Removal in Critically Ill Patients

**DOI:** 10.1186/2197-425X-3-S1-A19

**Published:** 2015-10-01

**Authors:** X Monnet, F Cipriani, L Camous, P Sentenac, M Dres, E Krastinova, N Anguel, C Richard, JL Teboul

**Affiliations:** Paris-South University Hospitals, Paris-South University, Medical Intensive Care Unit, Inserm UMR_S 999, Le Kremlin-Bicêtre, France; Paris -South University Hospitals, Paris-South University, Public Health Department, Le Kremlin-Bicêtre, France

## Objectives

To investigate whether haemodynamic intolerance to fluid removal during intermittent renal replacement therapy (RRT) in critically ill patients can be predicted by a passive leg raising (PLR) test performed before RRT.

## Methods

We included 39 patients in whom intermittent RRT with weight loss was decided: 13 with and 26 without intradialytic hypotension (IDH), defined as hypotension requiring a therapeutic intervention, as decided by the physician. Before RRT, the maximal increase in cardiac index (CI, pulse contour analysis) induced by a PLR test was recorded. RRT was then started.

## Results

CI significantly decreased faster in patients with IDH with a slope difference of -0.17 L/min/m^2^ per hour on average in comparison with those without. Ultrafiltration rate was similar in the two patient types. in patients with IDH, it occurred 120 min [interquartile range: 60-180 min] after onset of RRT. in the 26 patients with no IDH, the PLR test induced no significant change in CI. Conversely, in patients with IDH, PLR significantly increased CI by 15% [interquartile range: 11-36%]. PLR-induced increase in CI predicted intolerance to RRT with an AUC under the ROC curve of 0.89 (95% interval confidence: 0.75 to 0.97) (p < 0.05 from 0.50). The best diagnostic cut-off was 9%. It provided a sensitivity of 77%

(95% confidence interval: 46 to 95%), a specificity of 96% (80 to 100%), a positive predictive value of 91% (57 to 100%) and a negative predictive value of 89% (72 to 98%).

## Conclusions

The presence of preload dependence, as assessed by a PLR test before starting RRT with fluid removal, predicts that RRT will induce haemodynamic intolerance.

